# *Rotavirus* Diarrhea among Children in Taiz, Yemen: Prevalence—Risk Factors and Detection of Genotypes

**DOI:** 10.1155/2014/928529

**Published:** 2014-08-12

**Authors:** Abdulmalik Al-Badani, Leena Al-Areqi, Abdulatif Majily, Saleh AL-Sallami, Anwar AL-Madhagi, Mohammed Amood AL-Kamarany

**Affiliations:** ^1^Department of Pediatrics, Faculty of Medicine and Health Sciences, Taiz University, Taiz, Yemen; ^2^Department of Medical Microbiology, Faculty of Medicine and Health Sciences, Sana'a University, Sana'a, Yemen; ^3^The Yemeni-Swedish Hospital, Taiz, Yemen; ^4^Department of Pharmaceutical and Biomedical Sciences and Department of Pharmacy Practice, College of Clinical Pharmacy, Hodeidah University, P.O. Box 3114, Hodeidah, Yemen; ^5^Tihama Foundation for Drug Studies and Research, Hodeidah, Yemen

## Abstract

Diarrheal diseases are a great public health problem; they are among the most causes leading to morbidity and mortality of infants and children particularly in developing countries and even in developed countries. *Rotavirus* is the most common cause of severe gastroenteritis in infants and young children in both developed and developing countries. The purpose of this study was to determine the incidence rate of *Rotavirus* infection, its genotypes, and risk factors among children with diarrhea in Taiz, Yemen. 795 fecal samples were collected from children (less than 5 years old), suffering from diarrhea and attending the Yemeni-Swedish Hospital (YSH) in Taiz , Yemen, from November 2006 to February 2008. *Rotavirus* was detected by enzyme linkage immunosorbent assay (ELISA) on stool specimens of children. Genotypes of *Rotavirus* were characterized by reverse transcriptase-polymerase chain reaction (RT-PCR) and polyacrylamide gel electrophoresis (PAGE). The results showed that 358 (45.2%) were *Rotavirus*-positive and the most prevalent genotypes were G2P[4] (55%), followed by G1P[8] (15%). In addition, *Rotavirus* was found through the whole year; however, higher frequency during the summer season (53.4%) and lower frequency during the winter season (37.1%).

## 1. Introduction

Diarrheal diseases are a great public health problem that leads to morbidity and mortality of infants and children particularly in developing countries and even in developed countries [1]. In developing countries,* Rotavirus* is the most important cause of severe gastroenteritis among young children. Recent conservative estimates have indicated that 702,000 children die each year from* Rotavirus* disease and that up to 85% of these deaths occur in low-income countries [2, 3]. In many countries, the disease burden and epidemiology of* Rotavirus* are unknown because of the lack of adequate data or because there are no studies that have been conducted recently. In developed countries,* Rotavirus* remains a major clinical problem with 80% of children developing* Rotavirus* diarrhea in their first 3 years and the highest rates of illness occurring during the second year [4, 5]. In the United States of America (USA), approximately 20–60 deaths occur every year among children aged less than 5 years [6, 7] due to* Rotavirus*. Each year,* Rotavirus* infection results in the hospitalization of an estimated 70,000 children and 100 children died annually in the USA from complications of* Rotavirus* infection [7].

The aims of this study were to estimate the prevalence of* Rotavirus* infection, to detect the* Rotavirus* genotypes, and to determine the most cause factors of* Rotavirus* diarrhea among children under 5 years old in Taiz, Yemen. 

## 2. Materials and Methods

### 2.1. Study Area

Taiz is one of the largest governorates in Yemen, with an area of 10.000 km^2^. It is the highest populated governorate in Yemen; its population reaches up to 2.5 million inhabitants, of which 49% are males and 51% are females, organized in 23 districts. The total population of children under the age of five years is 540,000 according to the last national census made in 2004 by the Central Statistical Organization, Sana'a [8]. In the last few years, the city has suffered from a shortage of water resources and, as a result, the government constructed several dams; one of them is near the northern part of the city. The sewage system creates two major water collections in the north part of the city, 2-3 Km^2^ away from the dam. The water from the sewage system is also used in agricultural activities.

### 2.2. Study Design

A cross-sectional observational study was conducted by recruiting case-series of children who have diarrhea within the age group of less than 5 years of age. The study was done in the Yemeni-Swedish Hospital (YSH) and the National Center for Public Health Laboratories (NCPHL) in Taiz from November 2006 to February 2008. YSH was the only public hospital which admits children and practices mother health care in Taiz city.

### 2.3. Sample Size

With respect to the investigation's third object of risk factors for* Rotavirus*, recruiting 795 samples provided the study with more than 80% of power to detect an odd ratio of 2 or more, assuming that the prevalence of* Rotavirus* infection was 20%–30%. Children were selected by a random sample, by selecting every fifth child who came with diarrhea during the study period. Many patients were coming from the neighboring governorates, because of the long history of good reputation for being the first specialized hospital in Yemen. Seventy-three samples were positive for* Rotavirus*; they were sent for genotyping by PCR in the Naval Medical Research Unit 3 (NAMRU-3), Cairo, Egypt.

### 2.4. Ethical Issue

Patients, mothers, or child's guardian received a simple explanation for the aim of the study as an ethical issue. If they agreed, the sample was collected and an interview was conducted. Confidentiality of the collected data was achieved by keeping data record in a locked room with limited access to the research team only.

### 2.5. Samples Collection

Within two days after hospitalization, at least 4–8 mg of stool was directly collected and stored in a sterile plastic container. Samples were kept at 2–8°C, for a maximum of 8 days, until they were transported to the laboratory where they were stored at −20°C prior to analysis. Clinical information was extracted from the mothers or child's guardian. Information included the child's sex, age at admission, symptoms, hydration status, height, weight, and length of hospital stay.

### 2.6. Laboratory Analysis

#### 2.6.1. Detection of* Rotavirus* by Enzyme Linked Immunosorbent Assay (ELISA)

*Rotavirus* status was ascertained by ELISA (IDEIA Kit-Dako Ltd., Cambridgeshire, UK). This kit was employed by a polyclonal antibody prepared against the common antigen presented on* Rotavirus* VP6 (a major group specific protein). These antibodies were used in a solid phase sandwich type ELISA using a microplate containing 96 wells [9].

#### 2.6.2. Detection of* Rotavirus* Genotypes by Polymerase Chain Reaction (PCR)

*Rotavirus* genotypes were detected by reverse transcription-polymerase chain reaction (RT-PCR) on 45.2% of* Rotavirus*-positive samples. Two amplifications of RT-PCR were carried out for identification of* Rotavirus* gene 9 (G-genotyping) and gene 4 (P-genotyping). For determination of the G types, the viral RNA was extracted according to the instructions of QIAmp viral RNA Mini kit (Qiagen, USA) and specific primers were used for the VP7 genes of G serotypes and other specific primers for the VP4 genes of P serotypes. The extracted RNA was kept at −20°C until use [10, 11].

#### 2.6.3. Agarose Gel Electrophoresis

All PCR products were also examined by gel electrophoresis in 2% agarose gel and the* Rotavirus* genotypes were determined by the molecular weight of the amplicons. On the other hand, gel separating nucleic acid requires staining in order to be visualized; the stain contained ethidium bromide and then was viewed under ultraviolet illumination [10, 11].

### 2.7. Statistical Methods

Interview and laboratory data were analyzed using Epi Info. The descriptive analysis and the chi-square test were used to make comparisons among categorical variables. For all statistical analyses, a *P* value of less than 0.05 was considered statistically significant.

## 3. Results

### 3.1. Characteristics and Clinical History of Patients

#### 3.1.1. Age and Sex

Fecal samples were collected from 795 children (less than 5 years of age) diagnosed with diarrhea during the period from November 2006 to February 2008. All children had diarrhea for a period of 1-2 days before hospitalization.* Rotavirus* infection was detected in 45.0% using ELISA technique. The general characteristics of patients were shown in Table 1. The age range of patients was from 1 to 59 months and the median age of subjects was 9 months with range of 6–14 months, where 50% of cases occurred between 6 and 14 months.* Rotavirus* diarrhea was represented in the males as 61.5% while in the females it was represented as 38.5%. However, this difference was not statistically significant (*P* = 0.8). The higher frequency of* Rotavirus* diarrhea was in infants between 7 and 11 months (32.1%) and the lower frequency was in infants between 1 and 6 months (29.6%) (Table 2). Also, the incidence rate among the studied patients was 66.3% new cases of* Rotavirus* in age group per 100.000 inhabitants.

#### 3.1.2. Symptoms of* Rotavirus* Diarrhea

In addition, the clinical findings were diagnosed and recorded. Fever, vomiting, and diarrhea were the main symptoms (50.5%), while diarrhea alone was recorded in 10.7% (Table 3).

#### 3.1.3. Rehydration Therapy and Misprescription Intake Antibiotics

On the other hand, a total of 421 (53.0%) of 795 patients were admitted as inpatients for intravenous rehydration therapy; 196 patients of them had* Rotavirus* diarrhea, while 374 (47.0%) were seen in the outpatient ward receiving oral rehydration solution and 162 had* Rotavirus* diarrhea; this difference was not statistically significant (*P* = 0.39) (Table 2). Also, the results showed that about 60.5% of the patients infected with* Rotavirus* were given antibiotics empirically as a sort of treatment inside hospital by doctors (*P* = 0.001) and there was misprescription intake of antibiotics from the health worker (*P* = 0.025) (Table 4).

### 3.2. Seasonality of* Rotavirus* Diarrhea

**Figure 1:**
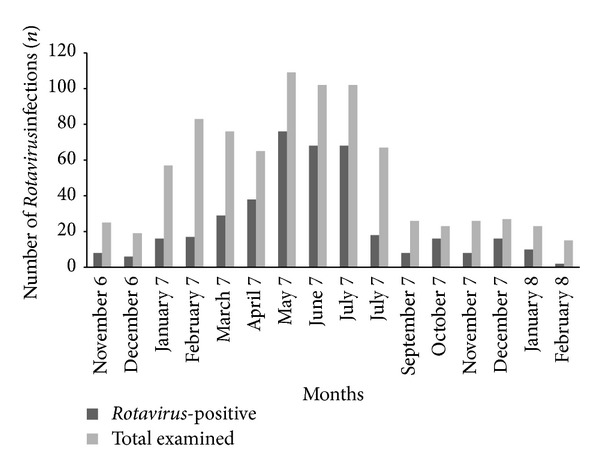
Monthly seasonality of* Rotavirus* diarrhea among children in Taiz, Yemen.

As for seasonal distribution,* Rotavirus* was found through the whole year. However, summer and winter seasons were significantly associated with* Rotavirus* infection; however, higher frequency during the summer season (140 cases, 53.4%, and *P* = 0.001) and lower frequency during the winter season (83 cases, 37.1%, and *P* = 0.005) (Table 5). In addition,* Rotavirus* exhibited peaks in May and June months (Figure 1).

### 3.3. Effect of Feeding on* Rotavirus* Diarrhea Outcome

In addition, the effect of breast-feeding versus mixed- and artificial-feeding on the outcome of the* Rotavirus* infection was studied in infants and young children; the results showed that the rate of the* Rotavirus* gastroenteritis was 67.5% of the breast-feeding and 32.5% of the artificial-feeding (Table 6).

### 3.4. Effect of Accommodation and Pattern Education on* Rotavirus* Diarrhea Outcome

The relationship between the* Rotavirus* infection and the type of house and education level of mothers was not found, while the* Rotavirus* infection and father educational level was statistically significant (*P* = 0.01). The source of water itself seems to have no effect in the transmission of* Rotavirus*. 39.0% of studied patients had animals at home; of those patients 41.3% had* Rotavirus* infection, but there was no statistical significance (*P* = 0.09). There was statistical significance (*P* = 0.026) between the presence of the latrine and its absence with infected* Rotavirus* patients (Table 7). Also, regarding the area of residence, only 7.0% of* Rotavirus* infections were from the semiurban area; 36.3% were from urban area, whereas 56.7% were from rural area. However, this difference was not statistically significant (*P* = 0.48) (Table 8).

### 3.5. Genotypes of* Rotavirus* Infection

**Figure 2:**
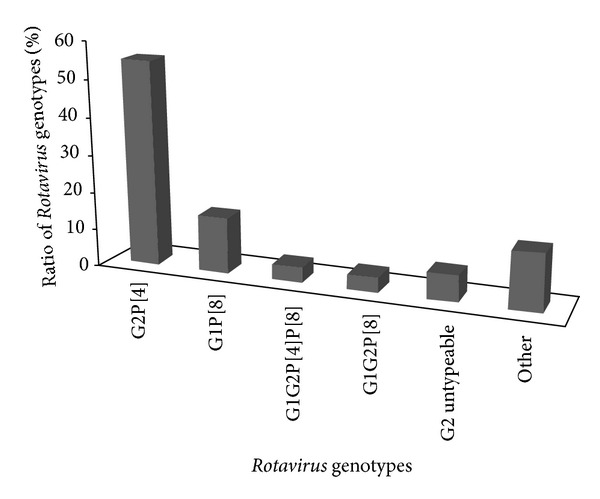
Common genotypes of* Rotavirus* diarrhea among children in Taiz, Yemen.

G and P genotypes of* Rotavirus* were detected in only 73 out of 358 (20%) by RT-PCR assay. G2P[4] (55.0%) was the predominant genotype, followed by G1P[8] (15.0%), also 4% of G1G2P[8], 4% G1G2P[4] P[8], and 7% of G2 untypeable and 15% of other genotypes (Figure 2). On the other hand, the seasonal variation of the most prevalent* Rotavirus* genotypes (G1P[8] and G2P[4]) was shown in Table 9.

## 4. Discussion

*Rotavirus* is a major cause of severe diarrhea of infants and young children. The present study showed that* Rotavirus* was the most common cause of diarrhea (45.2%) in infants and young children less than five years old in Taiz. Comparing to other studies carried out in different countries in the world, the present study results agreed with a study done by El Assouli et al. in Jeddah, Saudi Arabia, which stated that* Rotavirus* infection was 46% [12], while the higher rate of* Rotavirus* infection was recorded by Alicia et al. in Spain (55%) [13].

On the other hand, the lower percentages of* Rotavirus* infection were reported in many countries. The percentage of* Rotavirus* infection in infants and young children was 40% in Kuwait [14], 37% in both Turkey [15] and Erbil, Iraqi Kurdistan [16], 33% in Jordan [17], and 31% in Oman [18].

The rate of* Rotavirus* infection obtained in this study was more than those obtained in developed countries. For example,* Rotavirus* was found to be responsible for 10% of diarrhea in USA [19] and about 23.4% in infant diarrheal cases in the northwestern Ecuador [20]. The difference between the results may be due to the age of patients, duration of the study, and the methods used in detecting* Rotavirus*. In this study, two of the major global human* Rotavirus* genotypes (G2P[4] 55.0% and G1P[8] 15.0%) were detected and these genotypes were recorded in other studies in the neighboring Saudi Arabia [21]. In addition, in Yemen,* Rotavirus* infection was detected in 27% with genotypes that included G1P[8] (55%), G9P[8] (21%), and G2P[4] (12%) with G12 comprising 3% of strain types [22].

The infants who were less than one year old had the highest frequency of* Rotavirus* infection. Many other studies showed similar relationship between* Rotavirus* and age like the previous study in Yemen for children less than three years old who had diarrhea significantly higher than the young children [23], while this result agreed with other studies done in Egypt [24], Iran [25, 26], and Kuwait [14], where the higher frequency of* Rotavirus* was among infants less than 12 months. Also, the peak incidence of* Rotavirus* diarrhea in developing countries occurs between 6 and 11 months of age [27]. On the other hand, the difference between male and female in this study was not significant (*P* = 0.8), this result agreed with a previous study done in Hanoi [28].

During the period of the present study* Rotavirus* diarrhea occurred throughout the year, but the greatest number of* Rotavirus* diarrheas was identified in May and June; this result agreed with a study done in Jordan, where* Rotavirus* was more frequent during the summer months, June to August [29]. In addition,* Rotavirus* infection was reported in the cold, dry season in Tunisia [30]. This is also similar to the pattern seen in some African countries where* Rotavirus* infection had been recorded, such as Morocco [31, 32], Algeria [33], and Egypt [34], where epidemiological studies showed the same seasonal occurrence. This agreed with the result in this study which found that its peak was in the summer.* Rotavirus* infection has been called a winter disease in the temperate zones, whereas in tropical setting* Rotavirus* occurs through the whole year and this is in agreement with the current study.

The high prevalence of* Rotavirus* diarrhea among inpatients (54.7%) was not statistically significant in comparison with the prevalence of* Rotavirus* diarrhea in outpatients (45.3%) (*P* = 0.36) and this explains the role of* Rotavirus* in hospitalization of infants and children less than five years of age and the severity of diarrhea due to* Rotavirus* in practical medicine. Many other studies showed the burden of hospitalization due to* Rotavirus* infection.* Rotavirus* accounted for 30%–50% of diarrheal hospitalization in less than 5-year infants and children in England [35], 40% in diarrheal consultations in Argentina [36] and in New York,* Rotavirus* infection was reported in more than 30% of diarrhea cases in children less than 5 years of age [37].

The frequent association of fever and vomiting and the significant association of vomiting with* Rotavirus* diarrhea are not conclusive in the clinical diagnosis of* Rotavirus* infection, because such symptoms are constitutional and present with other causes of diarrhea. Nonetheless, the frequent vomiting with diarrhea leads to the risk of dehydration in* Rotavirus* infection more than other diarrheal infections and therefore increases the need for hospitalization.

The association between breast-feeding and* Rotavirus* infection may explain the poor personal hygiene practice. This link indicates their method of transmission, that is, the fecal-oral route. While breast-feeding protects against all-cause diarrhea in infants, no evidence shows that breast-feeding confers specific protection against viral gastrointestinal infection [38]. Several studies demonstrated that breast-feeding did not prevent acquisition of* Rotavirus* [39, 40]. Breast-feeding had no effect on reducing the severity of the symptoms of* Rotavirus* infection in this study. This was supported by other studies in Baghdad [41] and Yemen [23]. However, a beneficial effect of breast-feeding against* Rotavirus* infection was reported in other studies [42] which found that breast-feeding reduces the amount of vomiting in infants with* Rotavirus* infection [43] but the degrees of protection offered against* Rotavirus* infection vary in different populations [44].

There is no statistical significance between domestic animals and* Rotavirus* infection (*P* = 0.09). This result was supported by Al-Khasra et al. [23]. Socioeconomic status including level of education, type of house, source of water, and latrine was also studied as risk factor for transmission of* Rotavirus*. The latrine inside the home was significantly associated with* Rotavirus* infection but the other factors had no association. In a community-based survey carried out in two residential areas of different sanitary and socioeconomic conditions in Basra, Iraq, it was suggested that there is possibility of favoring* Rotavirus* transmission by poor personal hygiene, poor sanitation, and low educational level [45]. However, other studies did not prove the role of socioeconomic factors or sanitation except for the crowdedness factor [45].

*Rotavirus* was isolated from the nasopharyngeal secretion of two children out of thirty with upper respiratory tract infections [46]. In a recent study,* Rotavirus* was isolated from the oropharyngeal aspirates of 25 out of 89 infants with respiratory tract infections and 33 control patients without the disease [47]. This is in agreement with the current study, which shows that* Rotavirus* was detected in three patients out of four with upper respiratory tract infection (75.0%). This supports the other route of transmission of* Rotavirus*, which is respiratory droplets. Antibiotics were prescribed for 43.9% of all patients at the time of sample collection and for 45.4% of inpatients; 60.5% of patients with* Rotavirus* were given antibiotics empirically. Another study in Yemen indicated that antibiotics were used in 57.7% of children infected with* Rotavirus* [23]. This indicates the huge reliance on antibiotics treatment. This reflects the inappropriate diagnosis and lack of facilities of testing. Antibiotics should not be given routinely to children with diarrhea because they are ineffective and may lead to serious side effects [48].

In conclusion, this study revealed that* Rotavirus* is a major cause of diarrhea in infants and children in Taiz, Yemen. The incidence rate among the studied patients was found to be 66.3 new cases of* Rotavirus* in the studied age group per 100.000 inhabitants. The most predominant genotypes were G2P[4] (55.0%), followed by G1P[8] (15.0%);* Rotavirus* was found through the whole year with less-defined seasonal variation. However, the greatest number of the cases was identified in May and June of the year. The peak incidence of* Rotavirus* diarrhea in infants and young children in Taiz occurs between 7 and 11 months of age.

## Figures and Tables

**Table 1:** General characteristics of patients (*n* = 795).

Personal data	Number	Ratio
Sex		
Male	485	61.1
Female	310	38.9
Age (months)		
1–6	310	38.9
7–11	276	34.7
12–17	155	19.5
18–23	55	6.9%
24–60	80	10.1
Frequency of defecation/day (time)		
1–4	165	20.8
5-6	190	23.9
7–9	162	20.4
10+	278	34.9
Duration of diarrhea (day)		
1-2	334	42.0
3-4	343	43.2
5+	118	14.8
Hospitalization		
Inpatient	421	53.0
Outpatient	374	47.0

**Table 2:** Ratio of *Rotavirus* infection and characteristic of patients in Taiz, Yemen.

Personal data	Positive		Negative		*χ* ^2^	*P* value
	Number	Ratio	Number	Ratio		
Sex						
Male	220	61.5	265	60.6		
Female	138	38.5	172	39.4		
Age (months)						
1–6	106	29.6	123	28.1		
7–11	115	32.1	161	36.8		
12–17	70	19.6	85	19.5	3.74	0.29
18–23	43	12.0	12	2.8		
24–60	24	6.7	56	12.8		
Frequency of defecation/day (time)						
1–4	72	20.1	93	21.3		
5-6	77	21.5	113	25.9		
7–9	79	22.1	83	18.9	2.93	0.4
10+	130	36.3	148	33.9		
Duration of diarrhea (day)						
1-2	161	45.0	173	39.6		
3-4	159	44.4	184	42.1	9.45	0.009∗
5+	38	10.6	80	18.3		
Hospitalization						
Inpatient	196	54.7	225	51.5	0.84	0.39
Outpatient	162	45.3	212	48.5		

∗Significant.

**Table 3:** Symptoms of *Rotavirus* infection among children in Taiz, Yemen (*n* = 391).

Symptoms	*Rotavirus *				Total		Odd ratio	*P* value
	Positive		Negative			
	Number	%	Number	%	Number	%
Diarrhea alone	21	10.7	23	11.8	44	11.3	0.89	0.75
Vomiting alone	2	1.0	3	1.5	5	1.3	1.5	0.65
Diarrhea and vomiting	71	36.2	83	42.6	154	39.4	1.3	0.20
Diarrhea, vomiting, and fever	99	50.5	85	43.6	184	47.1	0.76	0.17
Upper respiratory tract infection	3	1.5	1	0.5	4	1.0	0.33	0.31

Total	196	100.0	195	100.0	391	100.0		

**Table 4:** Clinical history and *Rotavirus* diarrhea among children in Taiz, Yemen.

Type of house (*n* = 195)	*Rotavirus *	P value	Odd ratio	95% confidence interval
	Number			
Clinical history (*n* = 731)				
Health worker treatment	107	1.89	0.62	1.23–2.76
Costive medicines	22	1.19	0.03∗	0.62–2.25
Antibiotics	100	1.83	0.003∗	1.22–2.76
Oral rehydration	107	0.92	0.59	0.68–1.26
Public hospital treatment	163	0.78	0.93	0.54–1.59
Costive medicines	19	1.29	0.473	0.64–2.63
Antibiotics	107	2.2	0.0001∗	1.45–3.26
Oral rehydration solution	106	1.54	0.034	1.03–2.29

∗Significant.

**Table 5:** Seasonality of *Rotavirus* diarrhea among children in Taiz, Yemen.

Season	*Rotavirus *				Total		*χ* ^2^	*P* value
	Positive		Negative					
	Number	%	Number	%	Number	%		
Winter	83	37.1	141	62.9	224	10.44	0.02	0.88
Spring	100	44.6	124	55.4	224	12.57	0.17	0.67
Summer	140	53.4	122	56.6	262	17.61	0.03	0.86
Autumn	35	41.2	50	58.8	85	04.40	0.17	0.68

Total	358	45	437	55	795	45		

**Table 6:** Effect of feeding on outcome of *Rotavirus* diarrhea among children in Taiz, Yemen (*n* = 385).

Feeding if <2 years	*Rotavirus *		Odd ratio	*P* value	95% confidence interval
	Number	%			
Exclusively breast fed	34	17.8	0.57	0.026∗	0.35–0.94
Mixed with artificial formulas	35	18.3	0.78	0.38	0.46–1.34
Mixed with cow/goat milk	20	10.5	0.81	0.56	0.41–1.62
Mixed with weaning food	40	20.9	0.83	0.47	0.50–1.38
Mixed with artificial formulas/weaning food	15	7.9	1.05	0.89	0.51–2.19
Exclusively artificial formulas	15	7.9	0.77	0.52	0.35–1.69
Artificial formulas/weaning food	25	13.1	0.75	0.38	0.39–1.43
Mixed with cow/goat milk	7	3.7	1.28	0.63	0.46–3.50

Total	191	100.0			

∗Significant.

**Table 7:** Effect of accommodation on outcome of *Rotavirus* diarrhea among children in Taiz, Yemen.

Risk factors	*Rotavirus *		*P* value	Odd ratio	95% confidence interval
	Number	%			
Type of house (*n* = 390)					
Flat	20	10.3	0.83	0.93	0.49–1.78
House	175	89.7			
Hut, shack	0	00.0	0.08	1.02	0.94–1.97
Total	**195**	**100 **			

Education level (*n* = 466)					
Father (illiterate)	183	85.5	1.8	0.01∗	1.14–2.977
Total	**214**	**100.0**			
Mother (illiterate)	123	57.5	1.01	0.92	0.705–1.473
Total	**214**	**100.0**			

Latrine (*n* = 195)					
Latrine is available	191	98.0	3.4	0.026∗	1.09–10.65
Nonlatrine	4	2.0	1.1	0.62	0.62–2.28
Total	**195**	**100.0**			

Source of water (*n* = 466)					
Tap water	75	35.1	0.4	0.84	
Well	89	41.6	0.9	0.98	
Stream/spring	11	5.1	0.46	1.3	0.61–2.91
Water trunk	21	9.8	0.97	1.0	0.55–1.87
Others (treated water)	18	8.4	0.33	1.3	0.73–2.53
Total	**214**	**100.0**			

Animal at home (*n* = 472)					
Yes	76	34.9	0.09	0.7	0.49–1.05
Total	**218**	**100.0**			

∗Significant.

**Table 8:** Distribution of *Rotavirus* diarrhea among children according to the area of residence in Taiz, Yemen (*n* = 795).

Area of residence	*Rotavirus *				Total		*χ* ^2^	*P* value
	Positive		Negative					
	Number	%	Number	%	No.	%		
Rural	203	56.7	255	58.4	458	34.5	0.22	0.64
Urban	130	36.3	144	33.0	274	57.6	1.47	0.48
Semiurban	25	7.0	38	8.7	63	7.9	0.79	0.37

Total	358	100.0	437	100.0	795	100.0		

**Table 9:** Seasonality of *Rotavirus* genotypes among children in Taiz, Yemen (*n* = 44).

Season	*Rotavirus* genotypes				Total		*χ* ^2^	*P* value
	G1P[8]	%	G2P[4]	%	Number	%		
Winter	3	6.81	11	25	14	31.8	0.02	0.88
Spring	2	4.54	9	20.45	11	25.0	0.17	0.67
Summer	2	4.54	6	13.63	8	18.2	0.03	0.86
Autumn	3	6.81	8	18.18	11	25.0	0.17	0.68

Total	10	22.7	34	77.3	44	100.0		
